# Real‐world genomic testing and treatment patterns of newly diagnosed adult acute myeloid leukemia patients within a comprehensive health system

**DOI:** 10.1002/cam4.6442

**Published:** 2023-08-28

**Authors:** John C. Byrd, Jennifer L. Gatz, Cynthia Lim Louis, Alice S. Mims, Uma Borate, Ashley O. Yocum, Theophilus J. Gana, Amy Burd

**Affiliations:** ^1^ University of Cincinnati College of Medicine Cincinnati Ohio USA; ^2^ Regenstrief Institute Indianapolis Indiana USA; ^3^ The Ohio State University Columbus Ohio USA; ^4^ The Leukemia and Lymphoma Society Rye Brook New York USA; ^5^ Biopharmatech Consulting, Inc. Leesburg Virginia USA

**Keywords:** academic hospital, acute myeloid leukemia, age, chemotherapy treatment, community hospitals, genomic testing

## Abstract

**Background:**

We evaluated the frequency of genomic testing and treatment patterns by age category in patients with newly diagnosed (ND) acute myeloid leukemia (AML) treated in both academic‐ and community‐based health systems within a single Midwestern State.

**Methods:**

Retrospective analysis of data from the Indiana University Health System Enterprise Data Warehouse and two local cancer registries, of 629 patients aged ≥18 years with ND AML during 2011–2018. Primary outcome variables were, proportion of patients with genomic analysis and frequency of mutations. Chemotherapy was categorized as “standard induction” or “other chemotherapy”/targeted therapy, and hypomethylating agents.

**Results:**

Overall, 13% of ND AML patients between 2011 and 2018 had evidence of a genomic sequencing report with a demonstrated increase to 37% since 2016. Genomic testing was more likely performed in patients: aged ≤60 years than >60 years (45% vs. 30%; *p* = 0.03), treated in academic versus community hospitals (44% vs. 26%; *p* = 0.01), and in chemotherapy recipients than non‐therapy recipients (46% vs. 19%; *p* < 0.001). Most common mutations were *ASXL1*, *NPM1*, and *FLT3*. Patients ≥75 years had highest proportion (46%) of multiple (≥3) mutations. Overall, 31.2% of patients with AML did not receive any therapy for their disease. This subgroup was older than chemotherapy recipients (mean age: 71.4 vs. 55.7 years, *p* < 0.001), and was highest (66.2%) in patients ≥75 years.

**Conclusions:**

Our results highlight the unmet medical need to increase access to genomic testing to afford treatment options, particularly to older AML patients in the real‐world setting, in this new era of targeted therapies.

## Graphical abstract

A retrospective study of real‐world genomic testing and treatment patterns of newly diagnosed adult acute myeloid leukemia patients within a comprehensive health system in the Midwestern United States showed only 13% of patients had evidence of a genomic sequencing report and genomic testing was significantly more likely in younger patients =60 years versus older patients >60 years (45% vs. 30%, *p* = 0.03), in chemotherapy recipients than non‐therapy recipients (46% vs. 19%, *p* < 0.001), and in patients with academic treatment versus community hospital treatment (44% vs. 26%, *p* = 0.01). Overall, 31.2% of the patients did not receive any therapy, the subgroup was older than the chemotherapy recipients (mean age: 71.4 years vs. 55.7 years, *p* < 0.001) and was highest (66.2%) in patients =75 years; and highlights the unmet medical need to increase access to genomic testing in the real world, the need for continuing efforts to educate providers about the benefits of cytogenetic and genomic testing, and for considerations of models that afford opportunity to integrate academic and community practice to afford the best outcome for AML patients, particularly for older patients aged =75 years.

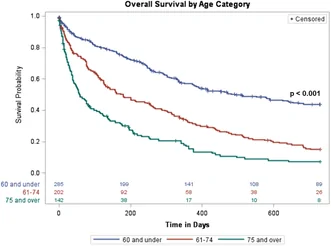

## 1 INTRODUCTION

Acute myeloid leukemia (AML), the most common form of acute leukemia diagnosed in adults yearly,^1^ is the leading cause of leukemia‐related mortality in the United States.^2^ It is estimated there will be 20,380 new cases of AML and 11,310 deaths from AML in the United States in 2023.^2^ Incidence of AML increases with age, with a median age at diagnosis of 69 years.^2^ With chemotherapy and hematopoietic stem cell transplantation (HSCT), AML is now cured in 35%–40% of adults aged ≤60 years and in 5%–15% of patients aged >60 years.^3^, ^4^ The prognosis in older patients unfit to receive intensive chemotherapy remains poor with a median overall survival (OS) of only 5–18 months based upon even most modern regimens. However, introduction of select targeted therapies over the past 5 years has afforded a small subset of patients who can have very durable remissions in the absence of either intensive therapy or allogeneic HSCT. This highlights the need to identify such patients to enable administration of such treatments in all settings of clinical practice.

Advances initially in cytogenetics, gene expression profiling, and most recently next‐generation sequencing (NGS) has led to the understanding that AML is a complex and heterogenous disease with multiple recurrent cytogenetic abnormalities and somatic mutations,^3^, ^5^, ^6^, ^7^, ^8^ which is translating into improved diagnosis, disease classification, refining risk stratification, novel therapeutic approaches, and improved clinical practice.^3^, ^9^, ^10^, ^11^, ^12^, ^13^, ^14^ These advances have facilitated development of novel therapies that target specific mutations in defined AML patient populations, allowing a personalized risk‐adapted approach to treatments.^14^, ^15^, ^16^ Consequently, these developments are increasing the adoption of genomic testing (GT) at diagnosis^12^ and the results of GT are already influencing the choice/timing of treatment in AML.^16^, ^17^ The feasibility of performing a comprehensive cytogenetic/molecular profiling (by NGS) in newly diagnosed (ND) AML patients aged ≥60 years within 7 days of sample receipt to prospectively assign treatment, based on the dominant clone, without increasing early death or adversely impacting OS, was recently demonstrated in a prospective study.^17^ However, to adequately describe the future impact of targeted therapies on outcomes, GT and treatment patterns in the real world need to be evaluated together. Indeed, in the community, alteration of practice pattern may be necessary to fully appreciate the benefit of any therapy appreciated in the context of a large center‐derived treatment approach for AML.

Retrospective studies have found that AML patients treated at high patient‐volume, academic or NCI‐designated cancer centers have improved outcomes than those treated at lower patient‐volume or smaller community practices or hospitals.^18^, ^19^, ^20^, ^21^, ^22^, ^23^, ^24^, ^25^, ^26^ However, little is known about treatment patterns and outcomes in a combined academic‐ and community‐based health system and few studies have evaluated GT patterns in academic hospitals/centers (AH/Cs) versus nonacademic/community hospitals (NA/CHs).^27^, ^28^ Therefore, we conducted a retrospective observational study with prospective aims in a real‐world cohort that included both AH/C and NA/CHs that collaborate with one another, within a comprehensive health system in the Midwest United States. Our patient dataset includes both metropolitan and rural populations, where we sought to evaluate GT and chemotherapy/targeted therapy treatment patterns by age category, and outcomes in ND AML patients from 2011 to 2018.

## 2 METHODS

### 2.1 Data source

**FIGURE 1:**
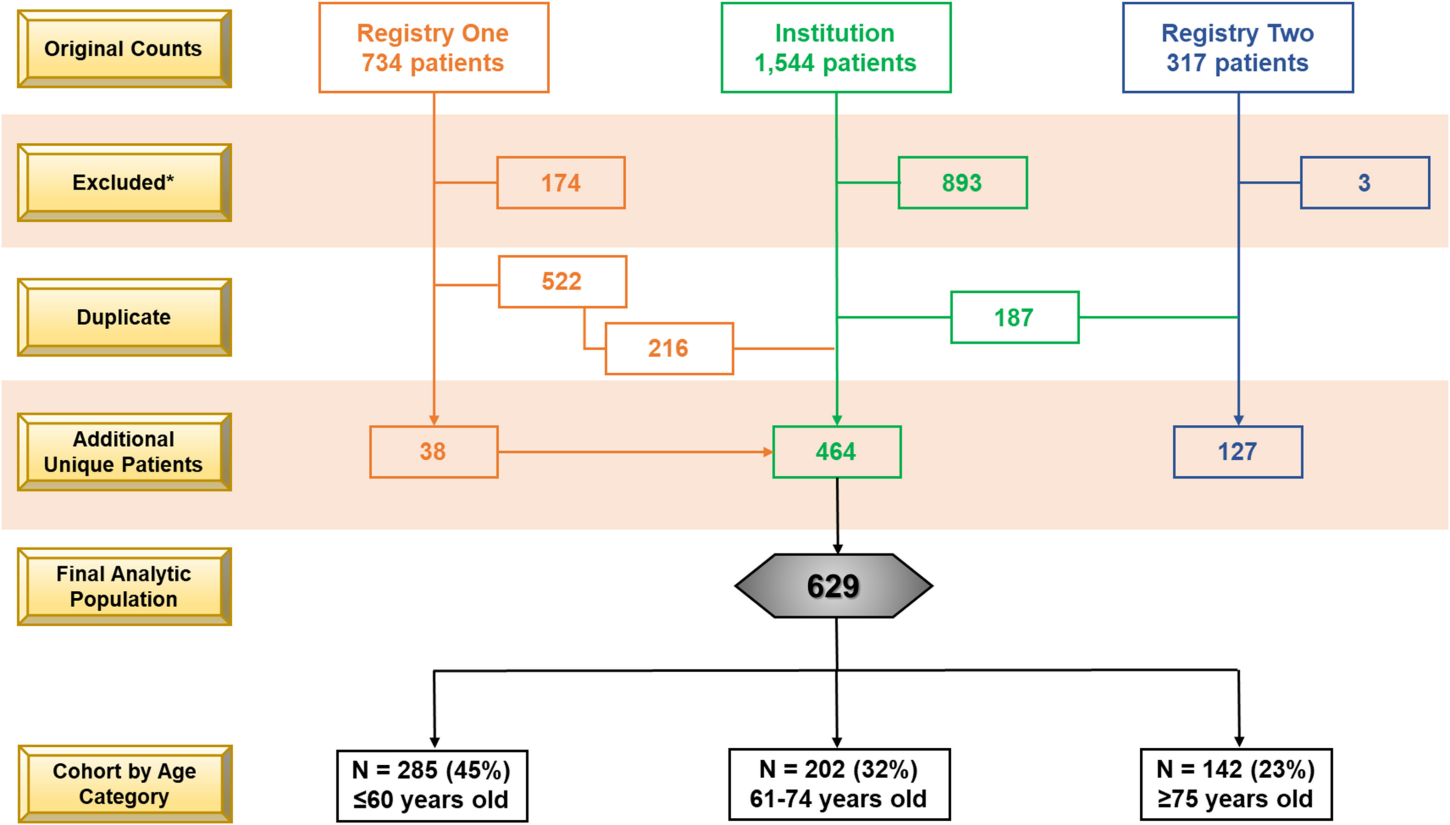
CONSORT flow diagram of the identification of the cohort of patients with newly diagnosed acute myeloid leukemia. The three named data sets containing information on patients from Indiana that were searched to identify potentially eligible patients aged ≥18 years with newly diagnosed acute myeloid leukemia between January 1, 2011 and June 30, 2018 who had at least one post‐diagnosis encounter within the IUHS for the study were: the IUH EDW (“Institution”), the IUH Tumor Registry (“Registry One”) and a database maintained by the Leukemia Program of the IU Simon Cancer Center (“Registry Two”). *Reasons for exclusion included: under 18 years old, non‐AML diagnosis, not newly diagnosed (diagnosed outside the institution and seeking consult or second opinion at the Institution), no follow‐up data available, and miscoded in the database. Note: Duplicate refers to process of removal of data that appeared in duplicates across all three data sources, Institution, Registry One and Registry Two. EDW, enterprise data warehouse; IUHS, Indiana University Health System; IUH, Indiana University Health.

The study was reviewed/approved by Indiana University (IU) Institutional Review Board. This was a retrospective observational study of the IU Health (IUH) Enterprise Data Warehouse (EDW) which is populated with data of patients treated within the IU Health System (IUHS). The largest health network in Indiana State, it saw over 600,000 inpatient days at 18 hospitals, 450,000 emergency department visits, and 2.6 million outpatient visits in 2017.^29^ We searched three datasets containing information on patients from Indiana to identify potentially eligible patients for the study: the IUH EDW (“Institution”), IUH Tumor Registry (“Registry One”) and a database maintained by the IU Simon Cancer Center (“Registry Two”). A total of 2595 patients with ND AML between January 1, 2011 and June 30, 2018 who had ≥1 post‐diagnosis encounter within IUHS were initially identified in the three data sources. The cohort selection is described in the CONSORT diagram (Figure 1). A final cohort of 629 patients was selected for the study. The date of diagnosis, abstracted from electronic health records or the registry, served as the index date.

### 2.2 Patient selection

Patients aged ≥18 years with ND AML between January 1, 2011 and June 30, 2018 were identified using the International Classification of Diseases, 9th Revision, Clinical Modification (ICD‐9‐CM) and 10th Revision (ICD‐10‐CM) diagnosis codes for AML, with a follow‐up period for each patient from date of initial diagnosis that ended with their death, 2 years after initial diagnosis or December 31, 2018, whichever came first. We excluded patients: aged <18 years, without AML (no blood/bone marrow involvement), not ND (diagnosed outside IUHS/seeking consult) by review of pathology reports, without follow‐up data, and with miscoded diagnosis by comparing the Institution data set patients' list versus Registry One and Registry Two lists.

### 2.3 Primary endpoints and variables analyzed

The primary outcome variables were, proportion of patients with GT and frequency of identified mutations. Genomic studies were identified by searching IUH Pathology database and sending a query to a commercial GT service, Foundation One (Foundation Medicine, Inc.). The reports were tabulated and reviewed to abstract identified mutations.

Patient demographics including age, gender, race, and ethnicity were obtained from the IUH EDW and cytogenetics by reviewing pathology and cytogenetic reports; patients were assigned risk using 2017 European LeukemiaNet (ELN) risk classification.^11^ Academic treatment (AH/CT) was defined as ≥2 inpatient or outpatient visits to IUH's AH/C on or within 2 months of the index date, combined with ≥2 post‐index encounters within the IUH EDW; patients with “non‐academic treatment” (NA/CHT) were seen only once at the AH/C or solely at the NA/CHs. We searched orders for FDA‐approved AML medications, obtained from National Cancer Institute website,^30^ to collect treatment information. Patients with administration records for both cytarabine and daunorubicin, idarubicin, or mitoxantrone were assigned the chemotherapy category “standard induction” (IC). Patients who received hydroxyurea only or with no records for chemotherapy were categorized as “no chemotherapy.” Patients with no records for both cytarabine and an anthracycline but had ≥1 record for cytarabine, an anthracycline or a hypomethylating agent (HMA; azacitidine, decitabine) or other agent (daunorubicin‐cytarabine liposomal, doxorubicin liposomal, gemtuzumab ozogamicin, tretinoin, tretinoin topical, arsenic trioxide, midostaurin, enasidenib, venetoclax) were categorized as “other chemotherapy” (OC). Clinical trial patients/investigational therapies identified during the study period were included in the appropriate chemotherapy category.

### 2.4 Overall survival

OS was determined from the index date to date of death from any cause with patients censored at 2 years from the index date or December 31, 2018, whichever occurred earlier. Dates of death were collected via the IUH EDW, Indiana Network for Patient Care,^31^ Social Security Administration Death Master File, or local online obituaries. If date of death was not documented, the last known date alive was used for censoring.

### 2.5 Statistical methods

Retrospective data were summarized using median (range) for continuous variables and frequencies/percentages for categorical variables. Statistical comparisons between groups were performed using Student's *t*‐test and chi‐squared test. We described the patterns of the variables by age category (≤60 years, 61–74 years or ≥75 years). Kaplan–Meier estimates were used to visualize OS. Kaplan–Meier OS curves were compared using the log rank test. A *p*‐value <0.05 was considered statistically significant. All summaries and analyses were performed using SAS software, version 9.4 (SAS Institute Inc.).

## 3 RESULTS

### 3.1 Study population

The demographics of the final cohort of 629 patients with ND AML and those with GT (*n* = 82) are shown in Table 1. Patients with GT were younger (median age 58.5 years) versus the entire cohort (median age 63.0 years).

**TABLE 1:** Demographics of identified cohort of patients with newly diagnosed acute myeloid leukemia and the subset with genomic data.

	All identified patients *N* = 629	Patients with genomic data *N* = 82
Age (years)		
Median (range)	63 (18–96)	58.5 (21–87)
Age at qualifying date, *n* (%)		
≤60 years old	285 (45%)	45 (55%)
61–74 years old	202 (32%)	24 (29%)
≥75 years old	142 (23%)	13 (16%)
Gender, *n* (%)		
Male	348 (55%)	48 (59%)
Female	281 (45%)	34 (41%)
Race, *n* (%)		
White	557 (89%)	76 (93%)
Black/African American	45 (7%)	2 (2%)
Others	9 (1%)	1 (1%)
Unknown	18 (3%)	3 (4%)
Ethnicity, *n* (%)		
Hispanic/Latino	14 (2%)	3 (4%)
Not Hispanic/ Not Latino	587 (93%)	76 (93%)
Unknown	28 (4%)	3 (4%)
Insurance type, *n* (%)		
Medicare	320 (51%)	30 (37%)
Medicaid	67 (11%)	3 (4%)
Commercial/self pay	24 (4%)	1 (1%)
Others (managed care, government, special contracts)	218 (35%)	48 (59%)
Patients by facility type, *n* (%)		
Academic hospital/outpatient center	390 (62%)	62 (76%)
Community hospitals	239 (38%)	20 (24%)

*Note*: Comparison of newly diagnosed AML patients who had genomic testing versus those who did not: Age: ≤60 years more likely to receive genomic testing versus ≥61 years (45% vs. 30%); *p* = 0.03. Insurance type (Medicare, Medicaid, commercial/self‐pay; Other) significantly associated with likelihood of receiving genomic testing; *p* < 0.001. Gender: males versus females were equally likely to receive genomic testing (37% vs. 37%); NS. *p*‐values are from chi‐squared tests; newly diagnosed AML patients used in the comparison are those with an index date on or after January 1, 2016–June 30, 2018.

Abbreviation: NS, not significant.

### 3.2 Academic/nonacademic facility visit

Of the 18 hospitals within the IUHS, there was one AH/C and one academic outpatient treatment center. A total of 390 patients (62.0%) received AH/CT and 239 patients (38.0%) received NA/CHT (Table S1). Chemotherapy recipients (IC or OC) were more likely to have received AH/CT than non‐recipients (75.1% vs. 33.2%, *p* < 0.001) and non‐chemotherapy recipients were more likely to have received NA/CHT than recipients (66.8% vs. 24.9%, *p* < 0.001).

### 3.3 Cytogenetics

Cytogenetics data were available for 500 patients (79.5%), and 48.8%, 24.6%, and 6.0% had 2017 ELN‐defined intermediate, adverse and favorable risk, respectively (Table S2). Proportionally, intermediate risk was similar and the highest across age categories. Favorable risk decreased with increasing age; no patient ≥75 years had favorable risk.

### 3.4 Genomics testing

Overall, 82 patients (13.0%) had evidence of a genomic sequencing report (Foundation One and IU Hematopathology Department) (Table 2); 75.6% were AH/CT recipients versus 24.4% NA/CHT recipients. Patients with GT showed a slight decreasing trend with increasing age (≤60 years: 15.8%; 61–74 years: 11.9%; ≥75 years: 9.2%).

**TABLE 2:** Number of mutations per patient and frequency of mutations during the study period by functional group and by age category.

Mutation	Total patients (*N* = 82)	Age category		
		≤60 years old (*N* = 45)	61–74 years old (*N* = 24)	≥75 years old (*N* = 13)
No mutation	18 (21.95%)	10 (22.22%)	4 (16.67%)	4 (30.77%)
1 mutation	18 (21.95%)	10 (22.22%)	7 (29.17%)	1 (7.69%)
2 mutations	22 (26.83%)	12 (26.67%)	8 (33.33%)	2 (15.38%)
3 mutations	14 (17.07%)	6 (13.33%)	3 (12.50%)	5 (38.46%)
4 mutations	8 (9.76%)	6 (13.33%)	1 (4.17%)	1 (7.69%)
5 mutations	2 (2.44%)	1 (2.22%)	1 (4.17%)	0 (0.00%)

Frequency of selected mutations				
Functional group mutation	Total number of mentions (*N* = 146)	Age category		
		≤60 years old (*N* = 81)	61–74 years old (*N* = 41)	≥75 years old (*N* = 24)
Methylation‐related group, *n* (%)				
DNMT3A	14 (9.59%)	11 (13.58%)	3 (7.32%)	0 (0.00%)
IDH2	10 (6.85%)	6 (7.41%)	2 (4.88%)	2 (8.33%)
TET2	10 (6.85%)	3 (3.70%)	4 (9.76%)	3 (12.50%)
IDH1	5 (3.42%)	2 (2.47%)	1 (2.44%)	2 (8.33%)
Total	39 (26.71%)	22 (27.16%)	10 (24.40%)	7 (29.16%)
Kinases group, *n* (%)				
FLT3^a^	18 (12.33%)	13 (16.05%)	4 (9.76%)	1 (4.17%)
KIT	5 (3.42%)	3 (3.70%)	1 (2.44%)	1 (4.17%)
JAK2	3 (2.05%)	3 (3.70%)	0 (0.00%)	0 (0.00%)
CSF1R	0 (0.00%)	0 (0.00%)	0 (0.00%)	0 (0.00%)
Total	26 (17.80%)	19 (23.45%)	5 (12.20%)	2 (8.34%)
NPM1, *n* (%)				
NPM1	20 (13.70%)	13 (16.05%)	6 (14.63%)	1 (4.17%)
Total	20 (13.70%)	13 (16.05%)	6 (14.63%)	1 (4.17%)
Chromatin remodeling group, *n* (%)				
ASXL1	20 (13.70%)	5 (6.17%)	8 (19.51%)	7 (29.17%)
Total	20 (13.70%)	5 (6.17%)	8 (19.51%)	7 (29.17%)
Tumor suppressors group, *n* (%)				
TP53	13 (8.90%)	6 (7.41%)	5 (12.20%)	2 (8.33%)
WT1	5 (3.42%)	4 (4.94%)	1 (2.44%)	0 (0.00%)
Total	18 (12.32%)	10 (12.35%)	6 (14.64%)	2 (8.33%)
RAS pathway group, *n* (%)				
NRAS	11 (7.53%)	7 (8.64%)	3 (7.32%)	1 (4.17%)
KRAS	3 (2.05%)	2 (2.47%)	1 (2.44%)	0 (0.00%)
PTPN11	2 (1.37%)	1 (1.23%)	1 (2.44%)	0 (0.00%)
Total	16 (10.95%)	10 (12.34%)	4 (12.20%)	1 (4.17%)
Transcription factors group, *n* (%)				
RUNX1	7 (4.79%)	2 (2.47%)	1 (2.44%)	4 (16.67%)
Total	7 (4.79%)	2 (2.47%)	1 (2.44%)	4 (16.67%)

^a^ FLT3‐TKD and FLTD‐ITD were combined to “FLT3.”

Before 2016, only four ND AML patients had GT and none had any of the selected mutations in Table 2. However, in the ND patients' subset with index dates between January 1, 2016 and June 30, 2018 (*n* = 210), GT increased to 37%, and patients ≤60 years were significantly more likely to receive GT than those ≥61 years (45% vs. 30%, *p* = 0.03); males and females were equally likely to receive GT (each 37%; *p* > 0.05); and GT frequency did not significantly vary by risk stratification. Chemotherapy recipients were significantly more likely to receive GT than non‐recipients (46% vs. 19%, *p* < 0.001), and AH/CT recipients were significantly more likely to receive GT than NA/CHT recipients (44% vs. 26%, *p* = 0.01).

Of the 82 patients with GT, 78.1% had ≥1 mutation and patients ≥75 years had the highest proportion (46.2%) of multiple (≥3) mutations (Table 2). Most frequent mutations were *ASXL1*, *NPM1*, and *FLT3*, and mutation frequencies were highest in patients: ≥75 years—*ASXL1*, *RUNX1*, and *TET2*; 61–74 years—*ASXL1*, *NPM1*, and *TP53*; and ≤60 years—*NPM1*, *FLT3*, *DNMT3A*, and *NRAS*. Most frequently mutated genes, by previously reported functional groups^5^ were methylation‐related, kinases, NPM1, and chromatin remodeling. Frequency of kinases was highest in patients ≤60 years, and chromatin remodeling and transcription factors were highest in patients ≥75 years.

### 3.5 Treatment/chemotherapy

#### 3.5.1 Nontreatment group

**FIGURE 2:**
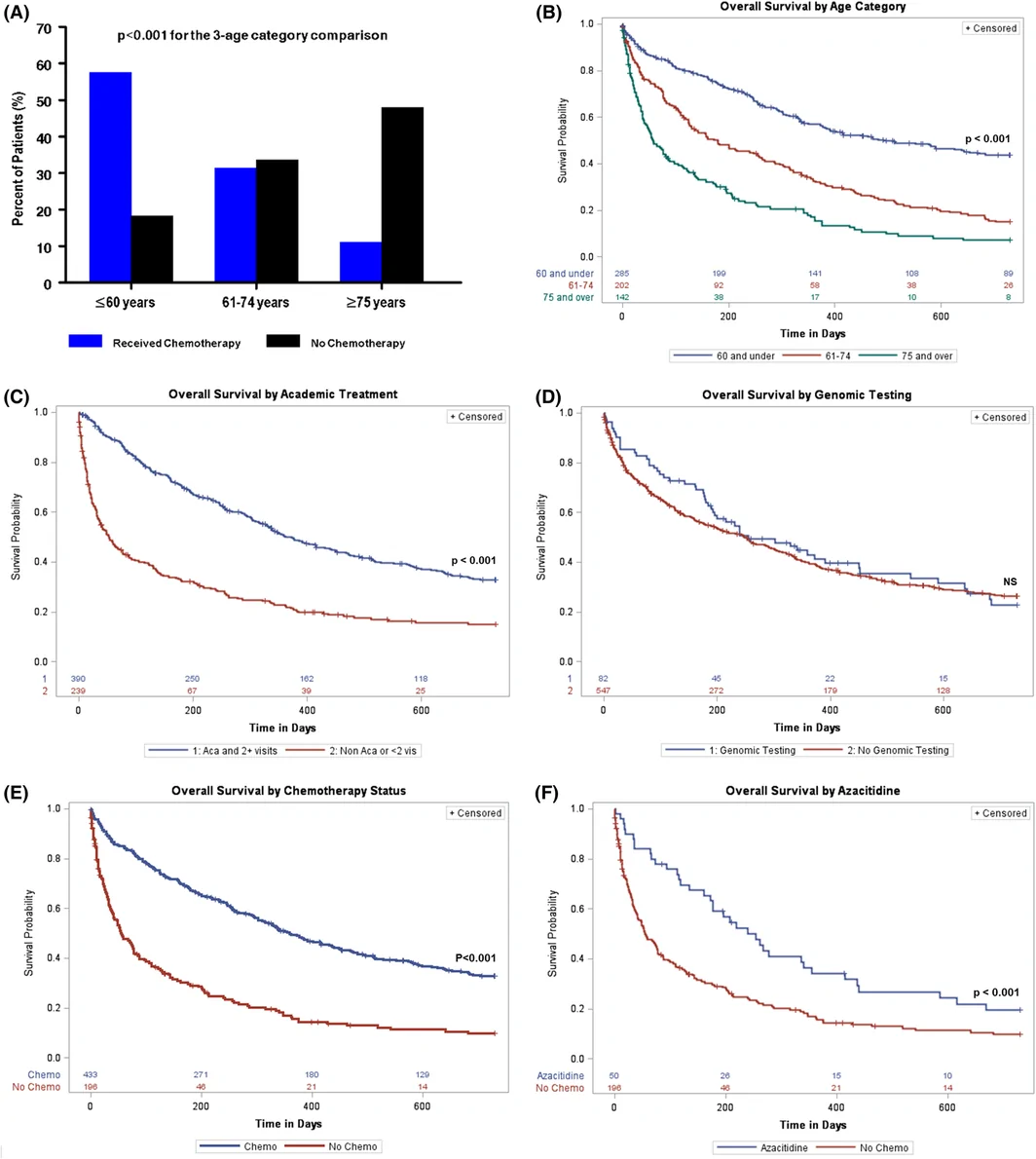
(A) Percentage of patients with newly diagnosed AML who received chemotherapy (standard induction or other) versus those who did not by age category. Patients who received chemotherapy were more likely to be in the younger age category than those who did not receive chemotherapy (≤60 years: 57.5% vs. 18.4%), and were less likely to be in the very old age category (≥75 years: 11.09% vs. 47.96%) (*p* < 0.001 for the 3‐age category comparison). (B) Kaplan–Meier overall survival (OS) curve for all newly diagnosed AML patients by age category: patients ≤60 years versus 61–74 years versus ≥75 years (*p* < 0.001 for 3‐age category comparison). (C) Kaplan–Meier OS curve for all newly diagnosed AML patients who received academic treatment versus those who received nonacademic/community hospital treatment (*p* < 0.001). (D) Kaplan–Meier OS curve for all newly diagnosed AML patients who received genomic testing versus those who did not (NS). Note: The OS probability among AML patients who did not receive genomic testing was unexpectedly high. This may be due to missing death data or misdiagnosis in this category. (E) Kaplan–Meier OS curve for all newly diagnosed AML patients who received chemotherapy (standard induction or other) versus those who did not receive any chemotherapy (*p* < 0.001). (F) Kaplan–Meier OS curve for all newly diagnosed AML patients who received azacitidine treatment versus those who did not receive any chemotherapy (*p* < 0.001). AML, acute myeloid leukemia; Chemo, standard induction chemotherapy or other chemotherapy recipients; NS, not significant. Chemotherapy categories are, standard induction = records for both cytarabine and anthracycline. Other chemotherapy = no records for cytarabine and anthracycline, but at least one record for cytarabine, anthracycline, a hypomethylating agent, or other agent. No chemotherapy = records for hydroxyurea only or no medication records at all. *p*‐value for the comparison of the percentages of patients who received chemotherapy versus those who did not by age category (A) was obtained using the chi‐squared test. *p*‐values for the comparison of the Kaplan–Meier OS curves were obtained using the log rank test.

Overall, 196 patients (31.2%) with mean age 71.4 years did not receive any therapy; of these, patients ≤60 years: 18.4%, 61–74 years: 33.7%, and ≥75 years: 47.96% (Table 3; Figure 2A). As reported in previous studies,^21^, ^22^, ^32^ proportion of non‐therapy recipients in this study increased with increasing age, with a striking 66.2% in patients ≥75 years.

**TABLE 3:** Frequency of chemotherapy and medication records in the cohort of patients with newly diagnosed acute myeloid leukemia and in those who have genomic records and a record of chemotherapy (*n* = 69) by age category.

Treatment/chemotherapy category	Total patients	Age category		
		≤60 years old	61–74 years old	≥75 years old
Patients who received treatment/chemotherapy				
No chemotherapy	196 (31.16%)	36 (12.63%)	66 (32.67%)	94 (66.20%)
Standard induction	341 (54.21%)	214 (75.09%)	112 (55.45%)	15 (10.56%)
Other chemotherapy	92 (14.63%)	35 (12.28%)	24 (11.88%)	33 (23.24%)
Total patients	629	285	202	142

Medication category	Total number of records	Age category		
		≤60 years old	61–74 years old	≥75 years old
Medication records^a^				
Cytarabine	357	221	119	17
Anthracyclines	367	235	116	16
Hypomethylating agents	75	16	28	31
Other agents	55	45	8	2
Hydroxyurea^b^	141	62	50	29

Treatment/chemotherapy category	Total patients	Age category		
		≤60 years old	61–74 years old	≥75 years old
Patients with genomic records who received treatment/chemotherapy (*n* = 69)				
No chemotherapy	13 (15.85%)	4 (8.89%)	5 (20.83%)	4 (30.77%)
Standard induction	56 (68.29%)	41 (91.11%)	13 (54.17%)	2 (15.38%)
Other chemotherapy	13 (15.85%)	0 (0.00%)	6 (25.00%)	7 (53.85%)
Total patients	82	45	24	13

*Note*: *Treatment/chemotherapy category*: Standard induction = records for both cytarabine and anthracycline. Other chemotherapy = no records for cytarabine and anthracycline, but at least one record for cytarabine, anthracycline, a hypomethylating agent, or other agent. No chemotherapy = records for hydroxyurea only or no medication records at all. *Medication category*: Anthracyclines = daunorubicin, idarubicin, mitoxantrone, and doxorubicin. Hypomethylating agents = azacitidine, and decitabine. Other agents = daunorubicin‐cytarabine liposomal, doxorubicin liposomal, gemtuzumab ozogamicin, tretinoin, tretinoin topical, arsenic trioxide, midostaurin, enasidenib, and venetoclax.

^a^ Patients can have more than one category of medication recorded.

^b^ Includes 29 patients with record of hydroxyurea only.

#### 3.5.2 Treatment group

Overall, 433 patients (68.8%), mean age 55.7 years, had records for chemotherapy (IC or OC/targeted therapy); their mean age was lower versus non‐therapy recipients (55.7 vs. 71.4 years, *p* < 0.001). Similar to previous studies,^22^, ^24^ chemotherapy recipients were more likely to be younger than non‐therapy recipients (≤60 years: 57.5% vs. 18.4%) and less likely to be in ≥75 years category (≥75 years:11.1% vs. 47.96%) (*p* < 0.001 for 3‐age category comparison) (Table 3; Figure 2A). Chemotherapy receipt did not significantly vary by gender, race or ethnicity, and decreased with increasing age. Of the patients with IC (54.2%), majority (62.8%) were ≤60 years (Table 3). Of the patients (14.6%) with OC/targeted therapy (includes HMAs), a higher proportion (35.9%) were ≥75 years. Cytarabine and anthracyclines records were similar within each age category, suggesting they were administered together.

In GT recipients versus the entire cohort: more patients received any chemotherapy (84.1% vs. 68.8%), higher proportion of patients ≤60 years received IC (91.1% vs. 75.1%), and fewer patients ≥75 years were non‐therapy recipients (30.8% vs. 66.2%) (Table 3).

### 3.6 Survival

The majority of patients (75.7%) died before the end of study period (June 30, 2018) and a higher proportion of older patients died (≥75 years: 88.7%; 61–74 years: 85.2% vs. ≤60 years: 62.5%) (Table S3). The OS probability for patients ≥75 years was lower versus those 61–74 years and ≤ 60 years (*p* < 0.001; Figure 2B). The median duration of follow‐up for all patients was 200 days (range: 1–730).

**FIGURE 3:**
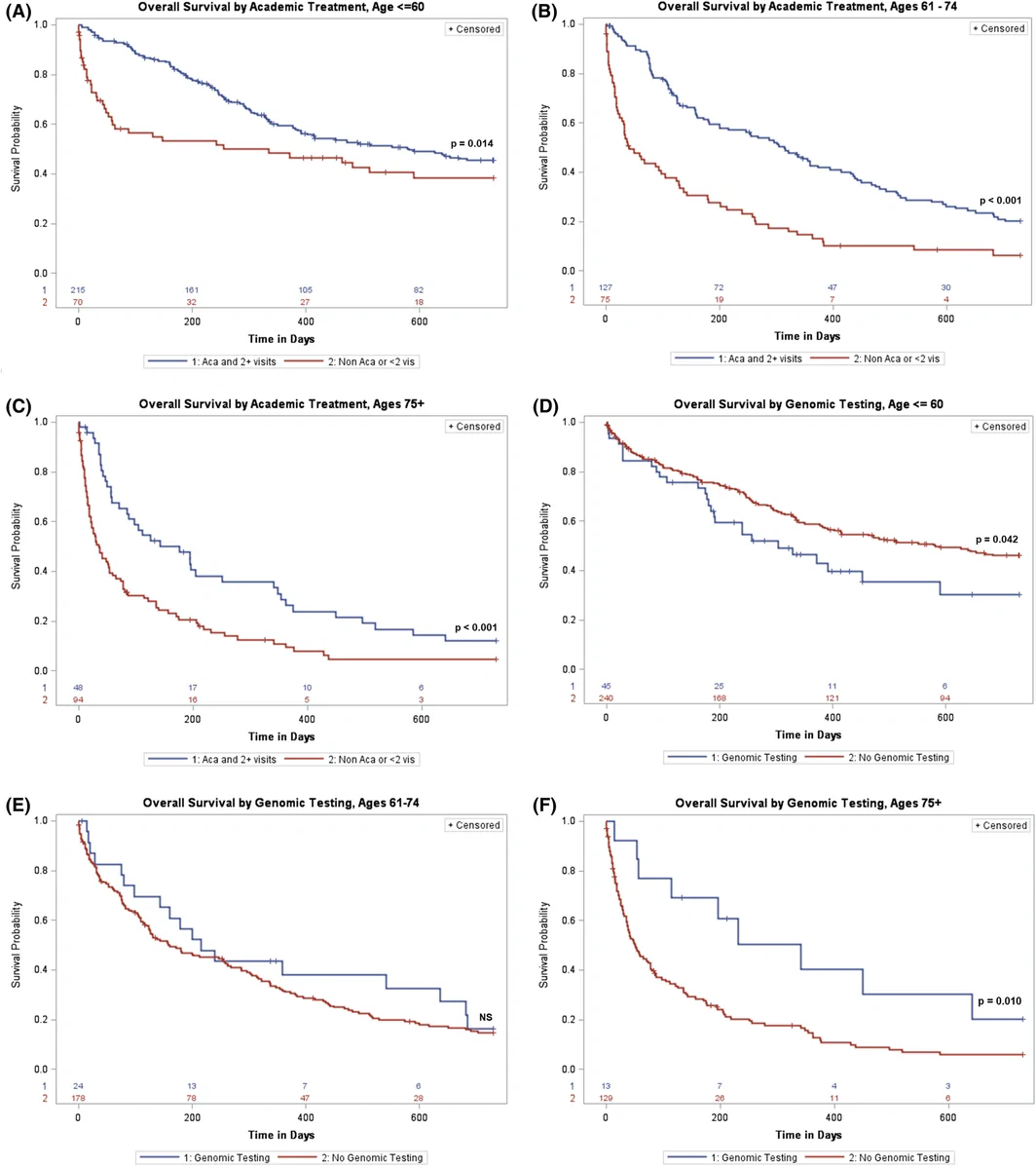
(A) Kaplan–Meier overall survival (OS) curve for newly diagnosed AML patients aged ≤60 years who received academic treatment versus those who received nonacademic/community hospital treatment (*p* = 0.014). Note: The OS probability for patients aged ≤60 years who did not receive academic treatment was unexpectedly high. This may be due to the low numbers of patients in this category after 1 year (*n* = 18 at 18 months post‐index), missing death data, or misdiagnosis. (B) Kaplan–Meier OS curve for newly diagnosed AML patients aged 61–74 years who received academic treatment versus those who received nonacademic/community hospital treatment (*p* < 0.001). (C) Kaplan–Meier OS curve for newly diagnosed AML patients aged ≥75 years who received academic treatment versus those who received nonacademic/community hospital treatment (*p* < 0.001). (D) Kaplan–Meier OS curve for newly diagnosed AML patients aged ≤60 years who received genomic testing versus those who did not. Note: The OS probability for patients aged ≤60 years who did not receive genomic testing was unexpectedly significantly higher versus those who received genomic testing (*p* = 0.042). This may be due to missing death data or misdiagnosis. (E) Kaplan–Meier OS curves for newly diagnosed AML patients aged 61–74 years who received genomic testing versus those who did not [NS]. (F) Kaplan–Meier OS curves for newly diagnosed AML patients aged ≥75 years who received genomic testing versus those who did not (*p* = 0.010). AML, acute myeloid leukemia; NS, not significant. *p*‐values for the comparison of the Kaplan–Meier OS curves were obtained using the log rank test.

The OS probability was significantly higher among AH/CT recipients (*p* < 0.001; Figure 2C) and by age category (*p* = 0.014; *p* < 0.001; and *p* < 0.001 for Figure 3A–C, respectively) versus NA/CHT recipients. The OS probability among GT recipients was similar versus non‐recipients (*p* = 0.552; Figure 2D), and OS was unexpectedly high among GT non‐recipients. The OS probability was unexpectedly significantly higher among GT non‐recipients ≤60 years versus recipients (*p* = 0.042; Figure 3D), was not significantly different among GT recipients 61–74 years versus non‐recipients (*p* = 0.374; Figure 3E), but was significantly higher in GT recipients ≥75 years versus non‐recipients (*p* = 0.010; Figure 3F).

The OS probability was significantly higher among chemotherapy recipients (*p* < 0.001; Figure 2C) and by age category (*p* = 0.028; *p* < 0.001; and *p* = 0.005 for Figure S1A–C, respectively), and among azacitidine recipients (*p* < 0.001; Figure 2F), versus the corresponding non‐therapy recipients.

## 4 DISCUSSION

Herein, we studied the patterns of GT and chemotherapy treatment concurrently in adult ND AML patients aged ≥18 years in both AH/C and NA/CHs that collaborate with each other, and by comparing NCCN‐defined^33^ younger patients ≤60 years versus older patients 61–74 years versus the very old ≥75 years. In contrast, the few previous studies of molecular genetic testing patterns in ND AML in academic and community hospitals did not specify patients' age range studied^27^ or studied older patients ≥55 years.^28^

Our results of this real‐world retrospective study of clinical data from IUHS EDW and two cancer registries during a 7.5‐year period, revealed only a low 13% of patients had evidence of a genomic sequencing report from 2011 to 2018. In 2016–2018, the likelihood of receiving GT was higher in patients: ≤60 years than >61 years (45% vs. 30%, *p* = 0.03), those with AH/CT than NA/CHT (44% vs. 26%; *p* = 0.01), and in chemotherapy recipients than non‐recipients (46% vs. 19%; *p* < 0.001). Of these, only AH/CT was found to be significantly associated with increased diagnostic molecular testing in previous studies^27^, ^28^; younger age reported as a factor previously was defined as 55 to <65 years.^28^ But in contrast to a previous study,^28^ we found GT receipt did not significantly vary by risk stratification, probably because our sample set was small.

Although majority (62%) of patients in our study received AH/CT, our GT rates at both AH/C and NA/CHs in 2016–2018 (44% vs. 26%; *p* = 0.01) are lower than the rates observed in previous studies (93% vs. 41%; *p* < 0.001^27^ and 84.3% vs. 70.2%; *p* < 0.001^28^). Furthermore, our 79.5% cytogenetics testing rate is lower than the 99.6%^27^ and 95.4%^28^ rates reported previously. It is possible that some cytogenetic and GT performed in this cohort were not available within the records used for this study. But the virtual lack of GT within the first 5 years of our study followed by an increase from 2016, may be due in part to availability/affordability based on initial high costs^16^ of GT which fell significantly by late 2015,^34^ or insurance reimbursement, or changing diagnostic practices following updated AML guidelines,^10^, ^11^, ^28^, ^35^ or growing awareness of how specific mutations impact survival and/or approval of new targeted therapies.^28^ However, our higher GT rate at the AH/C may be explained in part by improved access to GT and more rapid return of results.^26^

In our study, consistent with findings in previous studies of adult AML patients aged ≥18 years,^36^, ^37^, ^38^ most frequent mutations were *ASXL1*, *NPM1*, and *FLT3*; however, our frequencies are lower. By functional groups, most frequent mutations were in the methylation‐related, kinases and NPM1 groups, in concordance with previous studies.^36^, ^37^ Although total number of GT recipients in our study was small, the age distribution of the single mutations and functional groups are in concordance with findings of previous studies of AML patients ≥18 years as our study,^36^, ^37^, ^38^ suggesting real‐world matches the frequency occurrence in academic centers.

Our non‐chemotherapy treatment rate of 31.2% among all ND AML patients is similar to the 25% rate reported previously.^22^ However, our nontreatment rate is lower than the 43%–61.4% rates reported previously,^24^, ^32^, ^39^, ^40^ but, these included older (≥60 years)^39^ or elderly (≥65 years)^24^, ^32^, ^40^ patients with AML. Overall, 68.8% of patients received chemotherapy (IC or OC/targeted therapy/HMAs) and GT recipients were significantly more likely to have proceeded to chemotherapy as would be expected. Also, as reported previously, we found chemotherapy recipients were significantly more likely to have received AH/CT than non‐recipients,^21^, ^22^, ^24^ and to be younger (≤60 years) than older (61–74 and ≥75 years) patients.^22^, ^24^

The OS probability among GT recipients was not significantly different versus non‐recipients, which may be due to the unexpectedly high OS in GT non‐recipients. These results, the significantly higher OS in GT non‐recipients ≤60 years versus GT recipients, and the nonsignificant difference in OS in GT recipients 61–74 years versus non‐recipients, may be due in part to the small total number of GT recipients, missing death data or misdiagnosis. It is noteworthy that even during this study period, GT has been shown to influence the decision to proceed to HSCT versus receiving chemotherapy. Additionally, for molecular groups such as FLT3‐mutated AML, proceeding to treatment with midostaurin would be considered. As reported previously, OS for AH/CT recipients^19^, ^20^, ^21^, ^26^ was significantly higher versus NA/CHT recipients (*p* < 0.001) and in each age category (*p* = 0.014 to *p* < 0.001), a novelty in our study; similarly, OS for chemotherapy recipients^21^, ^22^, ^24^, ^32^, ^39^, ^40^ was significantly higher versus non‐recipients (*p* < 0.001) and in each age category (*p* = 0.028 to *p* < 0.001), also, a novelty in our study. The difference in outcome among AH/CT versus NA/CHT recipients likely is reflective of an often more fit patient that can travel to an AT, better expertise/experience of the AH/C treating AML versus the community, access to appropriate/complex supportive care, stratification toward transplantation when appropriate, and better access to clinical trials.^8^, ^26^

Our study is not without limitations. Databases and cancer registries do not capture access to NGS/GT, costs of testing or insurance reimbursement, which may determine availability/affordability, and influence GT rates. A survey of clinicians identified cost and lack of insurance reimbursement as the primary reasons for avoiding GT.^41^, ^42^ Factors such as performance status scores, bone marrow blast counts, patients' and physicians' preference which are used for making AML treatment decisions were not collected/analyzed. A previous report indicated that AML may be underreported in cancer registries.^43^ Nonetheless, our data suggest continuing efforts to educate providers about the benefits of cytogenetic and GT are needed as are considerations of models that afford opportunity to integrate academic and community practice to afford the best outcome for AML patients. Efforts particularly in patients aged ≥75 years are needed.

In conclusion, in ND AML patients, our results showed a higher rate of GT at AH/C than NA/CHs within a comprehensive health system. Furthermore, our results showed about a third of ND AML patients and two‐thirds of those ≥75 years are not receiving any therapy. In this new era of targeted therapies, our results highlight the unmet medical need to increase access to GT and chemotherapy treatment particularly to older patients in the real‐world setting, where most patients receive treatment.

## AUTHOR CONTRIBUTIONS

**John C. Byrd:** Conceptualization (equal); data curation (equal); formal analysis (equal); investigation (equal); methodology (equal); supervision (equal); writing – original draft (equal); writing – review and editing (equal). **Jennifer L. Gatz:** Conceptualization (equal); data curation (equal); formal analysis (equal); methodology (equal); writing – original draft (equal); writing – review and editing (equal). **Cynthia Lim Louis:** Project administration (equal). **Alice Mims:** Conceptualization (equal); data curation (equal); formal analysis (equal); investigation (equal); methodology (equal); writing – review and editing (equal). **Uma Borate:** Conceptualization (equal); data curation (equal); formal analysis (equal); investigation (equal); methodology (equal); writing – review and editing (equal). **Ashley O. Yocum:** Project administration (equal). **Theophilus J. Gana:** Writing – original draft (equal); writing – review and editing (equal). **Amy Burd:** Conceptualization (equal); data curation (equal); formal analysis (equal); funding acquisition (equal); investigation (equal); methodology (equal); supervision (equal); writing – original draft (equal); writing – review and editing (equal).

## FUNDING INFORMATION

Beat AML, LLC, a Division of the Leukemia & Lymphoma Society, provided funding for the study.

## CONFLICT OF INTEREST STATEMENT

John C. Byrd: *Research Funding* National Institutes of Health (NIH) grant R35 197734, UG1CA233338. *Stock and Other Ownership Interests* Vincerx Pharma Inc (a publicly traded company). *Consulting or Advisory Role* Novartis, Trillium, Astellas, AstraZeneca, Pharmacyclics, Syndax, Vincerx, Newave, and Orange Grove Bio, Kartos, and Kurome. *Honoraria*—AstraZeneca, Janssen. Jennifer L. Gatz and Cynthia Lim Louis: No disclosures. Alice S. Mims: *Consulting or Advisory Role* AbbVie/Genentech, Astellas Pharma, Novartis, Servier, Syndax, Ryvu Therapeutics, Zentalis, and DSMC—Jazz Pharmaceuticals and Daiichi Sankyo. Uma Borate: *Research Funding* AbbVie, Incyte, Jazz Pharmaceuticals and Pfizer. *Consulting or Advisory Role* Abbvie, Genentech, Agios, Novartis, Blueprint, Astellas, Takeda, Kura, and Servier. *Honoraria* RUNX1 Foundation. Ashley O. Yocum: *Employment* Leukemia & Lymphoma Society (LLS). *Research Funding* to LLS from—AbbVie, Agios Pharmaceuticals Inc. Amgen Inc., Astellas Pharma Inc., AstraZeneca, Boehringer Ingelheim International GmbH, Bristol‐Myers Squibb, Genentech Inc., Gilead Sciences Inc., ImmunoGen Inc., Jazz Pharmaceuticals, Johnson & Johnson, Novartis, Pfizer, Pharmacyclics, RTI Health Solutions, Shire and Takeda Pharmaceutical Company Ltd. Theophilus J. Gana: *Consulting or Advisory Role* Leukemia & Lymphoma Society. *Stock and Other Ownership Interests* Bausch Health. Amy Burd: *Employment* Leukemia & Lymphoma Society (LLS). *Research Funding* to LLS from—AbbVie, Agios Pharmaceuticals Inc. Amgen Inc., Astellas Pharma Inc., AstraZeneca, Boehringer Ingelheim International GmbH, Bristol‐Myers Squibb, Genentech Inc., Gilead Sciences Inc., ImmunoGen Inc., Jazz Pharmaceuticals, Johnson & Johnson, Novartis, Pfizer, Pharmacyclics, RTI Health Solutions, Shire and Takeda Pharmaceutical Company Ltd.

## Supporting information


Figure S1.

Tables S1–S3.

## Data Availability

The data that support the findings of this study are available on request from the corresponding author. The data are not publicly available due to privacy or ethical restrictions.
