# Supplementary material for: Real‐world genomic testing and treatment patterns of newly diagnosed adult acute myeloid leukemia patients within a comprehensive health system
Source: Cancer Med. 2023 Aug 28;12(17):18368–80. doi: 10.1002/cam4.6442 (PMC10524030; doi:10.1002/cam4.6442)
Supplement: Supplementary file 1 — Figure S1. [file CAM4-12-18368-s001.zip › cam46442-sup-0001-Figures1/revised_Byrd et al_Online-Only_Figure 1 Legend.docx]

**REVISED SUPPORTING INFORMATION: “ONLINE-ONLY” FIGURE LEGEND**

**“Online-Only” Figure Legend**

**Online-Only Figure 1 Legend:** (a) Kaplan–Meier overall survival (OS) curve for newly diagnosed AML patients aged ≤60 years who received chemotherapy (standard induction or other) versus those who did not receive any chemotherapy [p = 0.028]. Note: The overall survival probability for patients aged ≤60 years who did not receive any chemotherapy is unexpectedly high. This may be due to the low numbers of patients in this category after one year (n = 5 at 18 months post-index), missing death data, or misdiagnosis. (b) Kaplan–Meier OS curve for newly diagnosed AML patients aged 61-74 years who received chemotherapy (standard induction or other) versus those who did not receive any chemotherapy [p<0.001]. (c) Kaplan–Meier OS curve for newly diagnosed AML patients aged ≥75 years who received chemotherapy (standard induction or other) versus those who did not receive any chemotherapy [p = 0.005].

AML = Acute myeloid leukemia; Chemo = Standard induction chemotherapy or Other chemotherapy recipients.

Note: Chemotherapy Categories are: Standard Induction = Records for both cytarabine and anthracycline. Other Chemotherapy = No records for cytarabine and anthracycline, but at least one record for cytarabine, anthracycline, a hypomethylating agent, or other agent. No Chemotherapy = Records for hydroxyurea only or no medication records at all.

p-values for the comparison of the Kaplan-Meier OS curves were obtained using the log rank test.
