# Supplementary figures and images for: Real‐world genomic testing and treatment patterns of newly diagnosed adult acute myeloid leukemia patients within a comprehensive health system
Source: Cancer Med. 2023 Aug 28;12(17):18368–80. doi: 10.1002/cam4.6442 (PMC10524030; doi:10.1002/cam4.6442)

## Slide 1
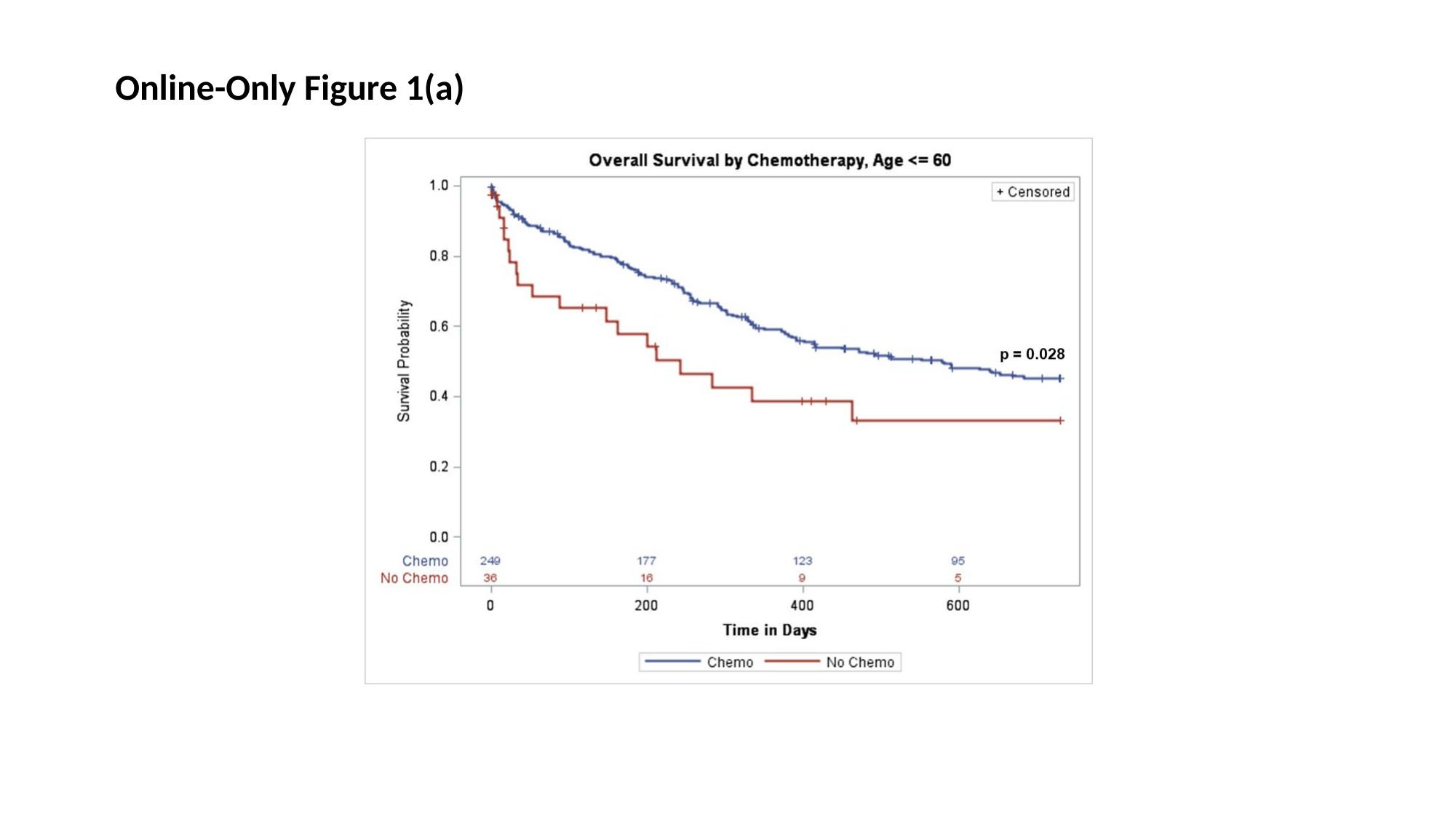

Online-Only Figure 1(a)

## Slide 2
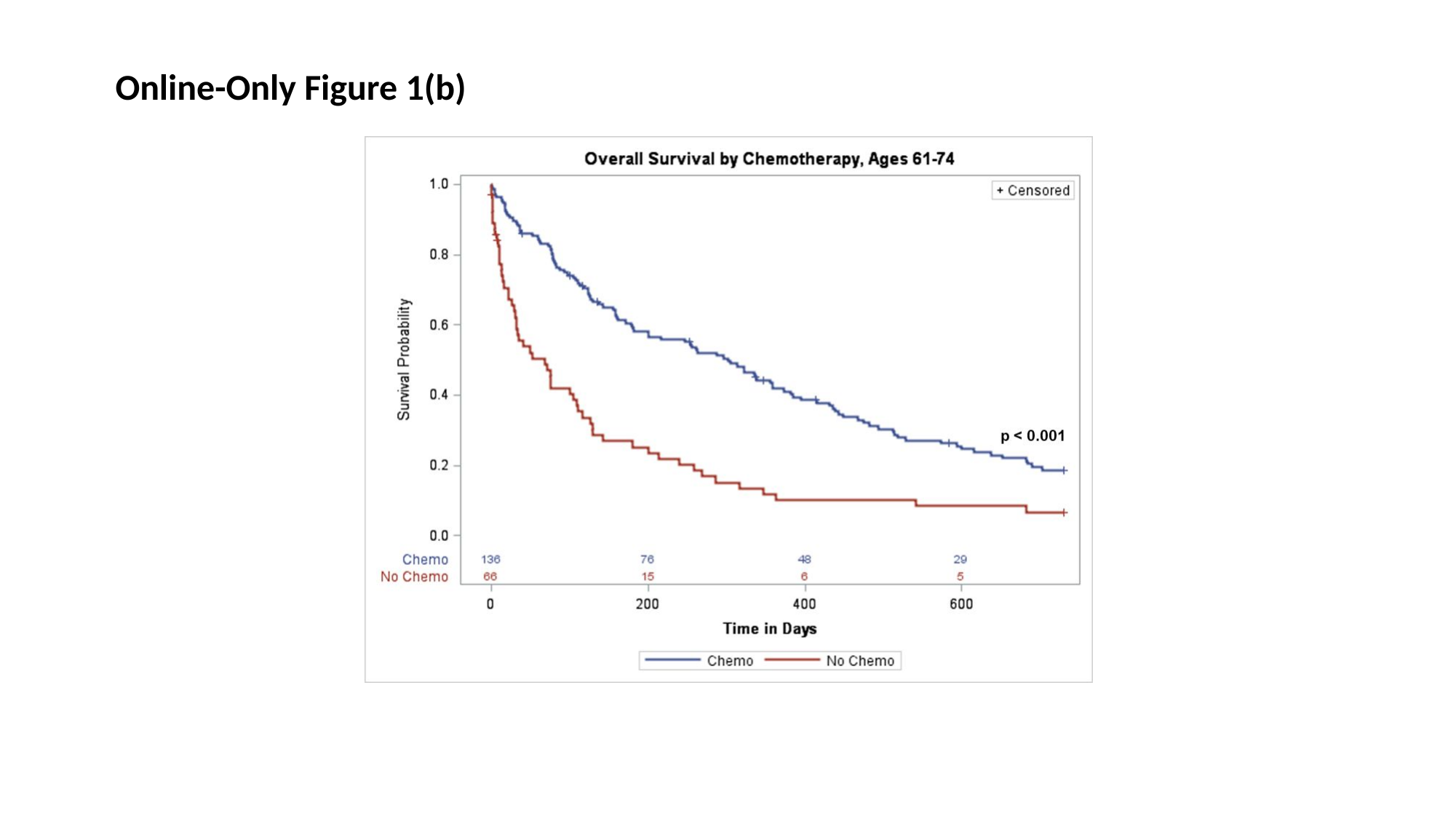

Online-Only Figure 1(b)

## Slide 3
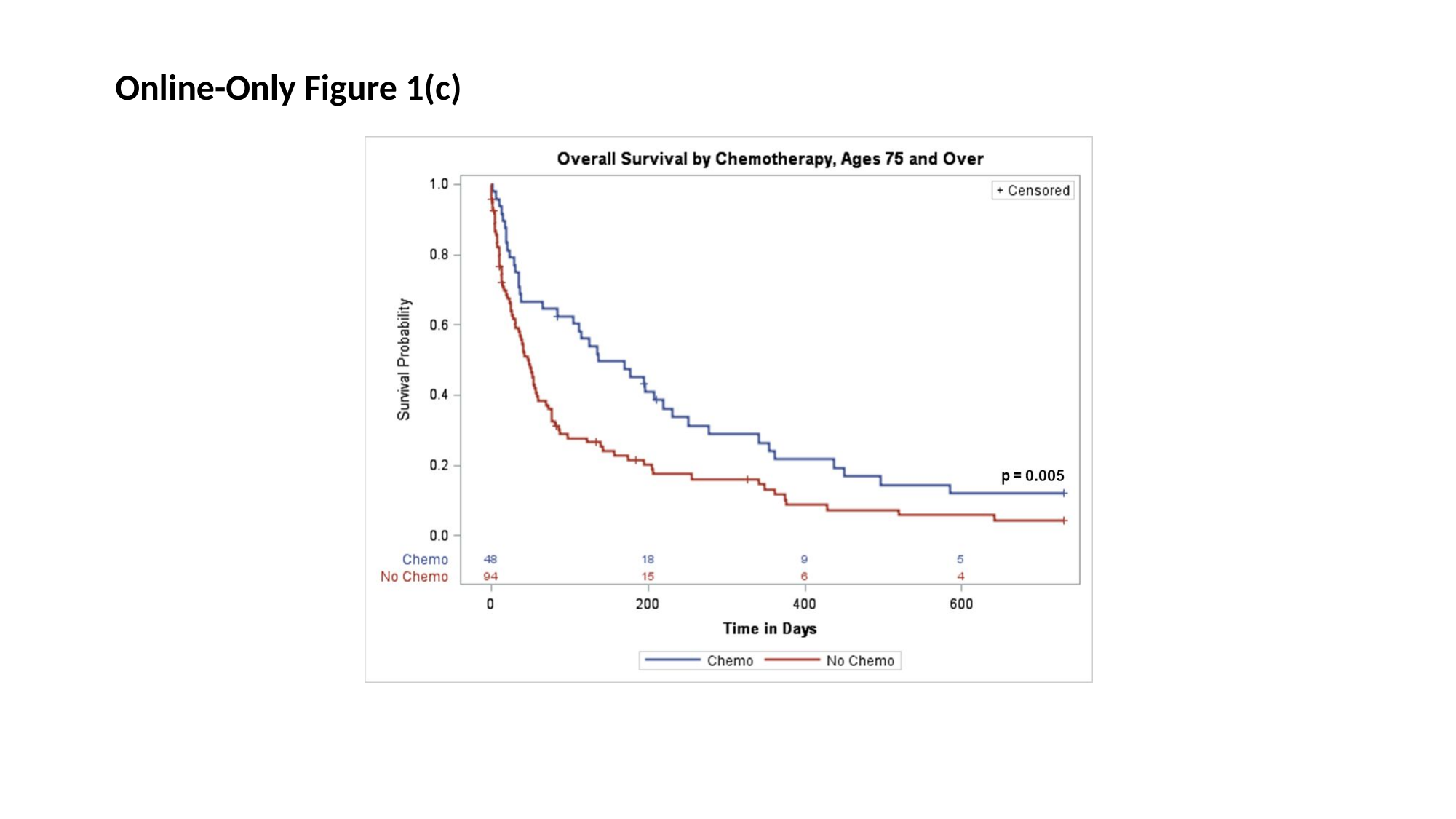

Online-Only Figure 1(c)

Supplement: Supplementary file 1 — Figure S1. [file CAM4-12-18368-s001.zip › cam46442-sup-0001-Figures1/cam46442-sup-0001-Onlineonlyfigure1a-1b-1c.pptx]
