# Supplementary material for: Real‐world genomic testing and treatment patterns of newly diagnosed adult acute myeloid leukemia patients within a comprehensive health system
Source: Cancer Med. 2023 Aug 28;12(17):18368–80. doi: 10.1002/cam4.6442 (PMC10524030; doi:10.1002/cam4.6442)
Supplement: Supplementary file 2 — Tables S1–S3. [file CAM4-12-18368-s002.docx]

**SUPPORTING INFORMATION: “ONLINE-ONLY” TABLES**

**Online-Only Table 1. Frequency of chemotherapy by facility type for the identified cohort of patients with newly diagnosed acute myeloid leukemia**

| **Treatment / Chemotherapy Category^†^** | **Total Patients** | **Academic Facility Treatment** | **Non-Academic / Community Hospital Treatment** |
| --- | --- | --- | --- |
| **No Chemotherapy** | **196 (31.16%)** | **65 (16.67%)** | **131 (54.81%)** |
| **Standard Induction** | **341 (54.21%)** | **272 (69.74%)** | **69 (28.87%)** |
| **Other Chemotherapy** | **92 (14.63%)** | **53 (13.59%)** | **39 (16.32%)** |
| **Total Patients** | **629** | **390** | **239** |

**^†^Treatment / Chemotherapy Category:** Standard Induction = Records for both cytarabine and anthracycline. Other Chemotherapy = No records for cytarabine and anthracycline, but at least one record for cytarabine, anthracycline, a hypomethylating agent, or other agent. No Chemotherapy = Records for hydroxyurea only or no medication records at all.

**Online-Only Table 2. Cytogenetics risk stratification of the identified cohort of patients with newly diagnosed acute myeloid leukemia and by age category**

| **Risk Category^†^** | **Total Patients** | **Age Category** | | |
| --- | --- | --- | --- | --- |
|  |  | **≤ 60 years old** | **61 - 74 years old** | **≥ 75 years old** |
| **Adverse** | **155 (24.64%)** | **63 (22.11%)** | **57 (28.22%)** | **35 (24.65%)** |
| **Intermediate** | **307 (48.81%)** | **139 (48.77%)** | **97 (48.02%)** | **71 (50.00%)** |
| **Favorable** | **38 (6.04%)** | **29 (10.18%)** | **9 (4.46%)** | **0 (0.00%)** |
| **Inadequate / Missing** | **120 (20.51%)** | **54 (18.95%)** | **39 (19.31%)** | **36 (25.35%)** |
| **Total Patients** | **629** | **285** | **202** | **142** |

**^†^**Risk stratification was based on the 2017 European LeukemiaNet classification.

Note: Overall, 500 patients had available cytogenetics data.

**Online-Only Table 3. Mortality of the identified cohort of patients with newly diagnosed acute myeloid leukemia during the study period and by age category**

|  | **Total Patients** | **Age Category** | | |
| --- | --- | --- | --- | --- |
|  |  | **≤60 years old** | **61 - 74 years old** | **≥75 years old** |
| **Alive** | **153 (24.32%)** | **107 (37.54%)** | **30 (14.85%)** | **16 (11.27)%** |
| **Dead** | **476 (75.68%)** | **178 (62.46%)** | **172 (85.15%)** | **126 (88.73%)** |
| **Total Patients** | **629** | **285** | **202** | **142** |
